# Supplementary material for: Causality Analysis with Information Geometry: A Comparison
Source: Entropy (Basel). 2023 May 16;25(5):806. doi: 10.3390/e25050806 (PMC10217183; doi:10.3390/e25050806)
Supplement: Supplementary file 1 [file entropy-25-00806-s001.zip › entropy-2285822-supplementary.pdf]

Supplementary Materials

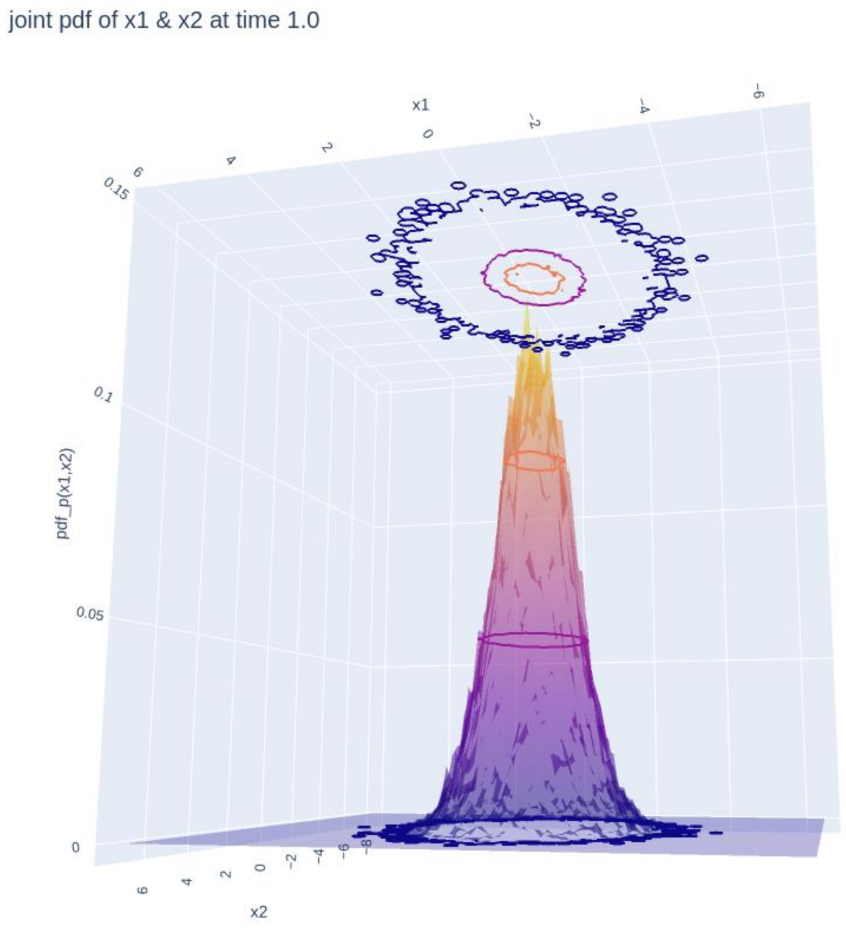

Figure S1. Estimation of the joint probability distribution via histogram.
